# Discovery of a fungal Tc toxin complex and functional mycoserpin through a novel two-by-two comparative genomics approach

**DOI:** 10.3389/fcimb.2026.1800461

**Published:** 2026-05-22

**Authors:** Zack Saud, Yujuan Luo, Martyn J. Wood, Ian Boostrom, Bruce J. MacLachlan, Richard J. Stanton, Chengshu Wang, Tariq M. Butt

**Affiliations:** 1 Department of Biosciences, College of Science, Swansea University, Swansea, Wales, United Kingdom; 2 Infection and Immunity, School of Medicine, Cardiff University, Cardiff, United Kingdom; 3 Key Laboratory of Insect Developmental and Evolutionary Biology, The Chinese Academy of Sciences (CAS) Center for Excellence in Molecular Plant Sciences, Shanghai Institute of Plant Physiology and Ecology, Chinese Academy of Sciences, Shanghai, China; 4 School of Life Science and Technology, ShanghaiTech University, Shanghai, China; 5 Institute of Molecular Biology and Biotechnology, Foundation for Research and Technology Hellas, Heraklion, Greece

**Keywords:** bioinformatics, biosynthetic gene cluster, comparative genomics, fungi, hypocreales, infection, serpin, Tc toxin

## Abstract

**Introduction:**

Fungi have been a rich source of pharmaceuticals such as antibiotics, immunosuppressants, and cholesterol-lowering drugs; however, their therapeutic potential remains largely untapped due to difficulties in culturing and elucidating the genetic basis of beneficial traits. Fungi contain ‘cryptic’ genes that are expressed under certain, and often obscure, growth conditions and can produce complex compounds that are difficult to synthesize economically. Developments in genome sequencing and DNA-synthesis technologies offer new opportunities to produce such compounds using biotechnological techniques, however, accurately identifying useful and novel genes, a prerequisite for such approaches, remains challenging.

**Methods:**

We present a novel ‘two-by-two’ comparative genomics pipeline for comprehensive gene analysis of selected fungal groups, enabling more confident identification of unique genes across the analyzed species. The approach compares gene sets from two strains of the same species with those from two strains of different species or families within a fungal order. Self-clustering orthologs that are unique to strains from the same species provide higher confidence in identifying species-specific proteins and help reduce noise from low-quality genome assemblies and gene prediction errors.

**Results:**

We validated our method in a well-studied fungal group, correctly identifying known species-specific genes and gene clusters, and discovering a novel functional myco-serpin and a fungal Tc toxin complex in *Metarhizium*.

**Discussion:**

Elucidating the genes underlying beneficial traits in fungi presents significant challenges, largely due to the unique and relatively complex aspects of their lifestyles. The two-by-two approach shows broad potential for fungal genome mining and bioprospecting, demonstrated by our discovery of the first fungal Tc toxin complex and a functional myco-serpin in *Metarhizium* (Hypocreales), and is applicable to other fungal orders such as Eurotiales and Xylariales. Furthermore, the two-by-two approach can be adapted to other organisms with genome architectures similar to fungi.

## Introduction

Fungi have played crucial roles in human industrial activities for millennia (Liu et al., 2018). The discovery of Penicillin marked the beginning of extensive exploration into fungal metabolites (Fleming, 1929; Alberti et al., 2017) that led to the development of further classes of fungal-derived pharmaceuticals such as; cyclosporine (Rüegger et al., 1976), an immunosuppressive agent, lovastatin (Alberts et al., 1980), a cholesterol-lowering drug, and various other antibiotics with differing modes of action, such as fusidic acid and cephalosporin (Newton and Abraham, 1955; Godtfredsen et al., 1962). Despite these achievements, the development of fungal therapeutics has lagged in comparison to other organisms for several reasons. Fungi exhibit intricate life cycles, encompassing the production of spores, mycelia, and fruiting bodies. Notably, the biosynthesis of bioactive compounds in fungi often occurs at specific developmental stages, with some metabolites being structurally complex and large. This inherent complexity can render their extraction and purification more cost-effective than chemical synthesis, as exemplified by the commercial production of cyclosporine (Anjum and Iram, 2015). Cultivating fungi and extracting metabolites often necessitates specialized techniques, with fewer than 5% of species having been successfully cultured to date (Kaeberlein et al., 2002). Additionally, large-scale extraction of useful compounds demands substantial investment in tailored bioreactors and monitoring systems for each species, making plant-based natural product discovery more financially attractive to pharmaceutical companies.

Fungi generally harbor more “cryptic” genes compared to other organisms that only activate in response to specific, often obscure, environmental cues or stressors, rendering them undetectable to transcriptomic, proteomic, and gene-knockout screening (Brakhage, 2013). This presents a significant opportunity for fungal natural product discovery through advanced comparative genomic approaches, as the presence of cryptic genes indicates that the biosynthetic potential of many cultivable fungi is likely much greater than currently recognized (Scherlach and Hertweck, 2021). With the advent of NGS and improved DNA synthesis technologies, novel approaches have been developed such as the “HEx” synthetic biology platform, wherein gene clusters are cloned from hard-to-culture fungi, refactored, and optimized to express in *Saccharomyces cerevisiae*, a traditional ‘cell factory’ for pharmaceutical production due to its simple growth requirements and rapid proliferation (Harvey et al., 2018). The compact genomes of Ascomycota fungi (30–40 Mbp) (Mohanta and Bae, 2015) make full genome sequencing and assembly both affordable and feasible, as highlighted by the recent surge of fungal genome submissions to databases like GenBank, and we have completed telomere-to-telomere assemblies, including mitochondrial genomes, for Ascomycota fungi for under $1000 (Saud et al., 2021). While some fungal genome sequences are high-quality, telomere-to-telomere assemblies or contain some complete chromosomes, many are assembled solely using short-read sequencing technologies and are thus more fragmented. The impact of this fragmentation on gene and biosynthetic gene cluster prediction accuracy remains unclear.

Despite the opportunities that have arose from technological advancements, there remains challenges as the bioinformatics analyses employed to identify functionally relevant fungal genes lack standardization and entail inherent complexity, often utilizing homology-based searches of biosynthetic gene cluster databases, likely biasing against discovery of completely novel clusters (Scherlach and Hertweck, 2021). Fungi boast higher gene densities relative to plants and animals (Galagan et al., 2005), with variation of gene sets being observed, even between closely related strains. This exacerbates the likelihood of false positive and negative discovery rates at the bioinformatics stage, especially given the intrinsic variance that can occur from differing genome assembly pipelines (sequencing technologies, read pre-processing strategies, assembler algorithm usage, polishing methods) and gene prediction approaches (e.g. *ab-initio*, homology-based, or RNA-seq guided) (Ter-Hovhannisyan et al., 2008). Given the costs and low throughput of designing, cloning, and expressing genes in heterologous hosts (Harvey et al., 2018), bioinformatics tools that confidently prioritize candidate genes are essential. There is a need for simple methods that can accurately identify strain-specific, species-wide, or genus-wide genes across diverse fungal genomes, achieving high confidence despite inherent genomic error rates. To address this need, we present the “two-by-two” approach, which compares gene sets from two strains of the same species with those from two strains of different species or families within a fungal order. Self-clustering orthologs between strains of the same species provide higher confidence in identifying species-specific proteins and help reduce noise from low-quality genome assemblies and gene prediction errors in comparison to comparing species using only a single representative strain. While pan-genome analysis examines all strains of a species, the two-by-two approach compares only two strains to identify species-specific genes, making it more suitable for organisms with less abundant genomic data.

We conducted a proof-of-concept study on Hypocreales fungi, an order with several well-studied species and multiple genomes of varying quality, including some telomere-length assemblies, available in GenBank. Notable members of Hypocreales include *Trichoderma* spp., widely used in agriculture as biocontrol agents against plant pathogens (Woo et al., 2023); *Cordyceps* spp., entomopathogens valued in traditional medicine, with compounds under study for immunomodulatory and anticancer effects (Tuli et al., 2014); *Beauveria bassiana*, a common pest management agent (Zimmermann, 2007); and *Metarhizium* spp., also used in pest control, with conidia that have been demonstrated to be larvicidal against disease-vectoring mosquitoes (Butt et al., 2013) and shown to produce compounds with immunomodulatory (Lv et al., 2020) and anticancer (Dornetshuber-Fleiss et al., 2013) potential. Many genes involved in producing bioactive compounds in these fungi are well-characterized, allowing effective benchmarking of our approach. Additionally, the diverse ecological niches occupied by these closely related fungi enable assessment of the hits by plausibility. The test group includes five closely related species from Cordycipitaceae, two distantly related species from Clavicipitaceae, and one from Hypocreaceae, allowing evaluation of the approach in distinguishing unique genes across varying levels of relatedness.

Using our two-by-two approach, we identified two novel proteins in *Metarhizium brunneum*: a functional serpin-like protein with homology to Drosophila melanogaster serpin 42Dd, an immune modulating protein, and a fungal Tc toxin complex with structural homology to bacterial Tc toxin complexes. Our findings provide a valuable reference for genome mining within the Hypocreales order and demonstrate that this analysis method can be applied to other fungal orders or organisms with high gene densities and significant interspecies gene variation, where potentially useful but under-characterized gene products are present.

## Results

### Genome attributes of species used in analyses

Genomes were chosen from the Hypocreales families *Cordycipitaceae*, *Clavicipitaceae*, and *Hypocreaceae*, utilizing criteria such as genome quality as assessed by BUSCO score and assembly N50, giving preference to assemblies that were less contiguous.

A total of 16 genomes comprising two strains from each species within three families in the order Hypocreales were subjected to orthologous protein and biosynthetic gene cluster analyses (**Table 1**). 6 of the genomes in the group had all 7 chromosomes sequenced telomere length, and these included; both strains of *Trichoderma reesei*, *Cordyceps javanica* isolate Apopka 97, *Cordyceps militaris* isolate ATCC 34164 (Kramer and Nodwell, 2017), *Metarhizium brunneum* isolate ARSEF 4556 (Saud et al., 2021), and *Epichloe festucae* isolate FL1. We performed gene predictions for species with genbank entries that lacked any gene information and the included; *Cordyceps fumosorosea* grCorFumo1 (10158 genes), *Cordyceps cateniannulata* FRD 24 (10850 genes), *C. cateniannulata* MBC 234 (11452 genes), *E. festucae* RoseCity (6979), *T. reesei* CBS999.97 (9196 genes), *T. reesei* QM6a (9298 genes).

**Table 1:** Assembly and annotation statistics for Hypocreales fungi used in this study.

Species	Family	Isolate	Assembly accession	Release year	Total length	Scaffolds (contigs)	N50	Full chromosomes (plasmids)	Predicted proteins	Protein busco (N = 4494)
*Cordyceps javanica*	*Cordycipitaceae*	Apopka 97	GCA_051103115.1	2024	35.14	–	–	7 (1)	10469	96.90%
*Cordyceps javanica*		**IJ2G**	GCA_006981975.1	2019	34.97	173 (658)	1822545	0	11142	94.40%
*Cordyceps fumosorosea*		**ARSEF 2679**	GCA_001636725.1	2016	33.49	430 (685)	872179	0	10061	95.60%
*Cordyceps fumosorosea*		grCorFumo1	GCA_963580265.1	2023	34.32	–	–	9 (1)	*10158	94.00%
*Cordyceps militaris*		ATCC 34164	GCA_008080495.1	2017	33.62	–	–	7 (0)	9287	92.40%
*Cordyceps militaris*		**CM01**	GCA_000225605.1	2011	32.27	32 (597)	4551492	0	9651	95.90%
*Cordyceps cateniannulata*		**FRD 24**	GCA_028828415.1	2023	33.63	0 (16)	4260819	0	*10850	96.90%
*Cordyceps cateniannulata*		MBC 234	GCA_030411495.1	2023	33.99	0 (1,338)	104394	0	*11452	95.90%
*Beauvaria bassiana*		ERL836	GCA_010099065.1	2020	35.40	0 (14)	3988868	0	10399	91.30%
*Beauvaria bassiana*		JEF-350	GCA_021365345.1	2022	35.70	0 (8)	5649914	0	7829	95.50%
*Metarhizium brunneum*	*Clavicipitaceae*	ARSEF 4556	GCA_013426205.1	2020	37.80	–	–	7 (1)	11420	99.10%
*Metarhizium brunneum*		**ARSEF 3297**	GCF_000814965.1	2015	37.10	92 (180)	1825569	0	10689	97.10%
*Epichloe festucae*		**Fl1**	GCA_003814445.1	2018	35.00	–	–	7 (1)	7034	87.40%
*Epichloe festucae*		RoseCity	GCA_016859245.1	2021	31.00	0 (19)	2735662	0	*6979	84.50%
*Trichoderma reesei*	*Hypocreaceae*	CBS999.97	GCA_016806755.1	2021	34.30	–	–	7 (0)	*9196	98.70%
*Trichoderma reesei*		QM6a	GCA_002006585.1	2017	34.90	–	–	7 (0)	*9298	98.00%
		**Bold Isolate** = RefSeq						* = Gene predictions performed in this study		

Bold isolate names indicates that the assembly is the reference genome sequence for that species. An asterisk in front of the predicted proteins number indicates that the gene prediction was performed in this study.

### Two-by-two biosynthetic gene cluster analysis of hypocreales

**Figure 1:**
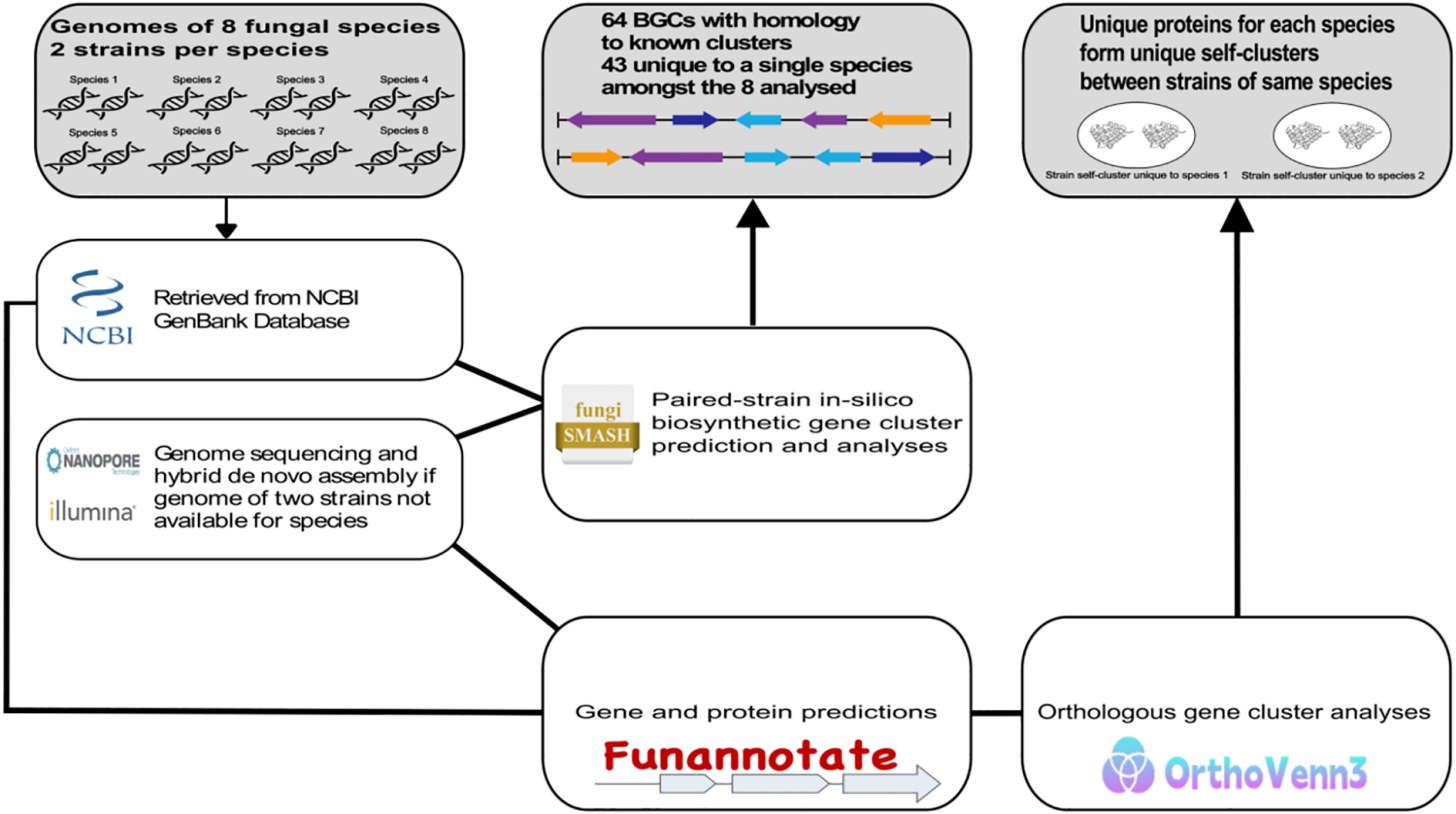
Overview of the two-by-two approach. Instruments, bioinformatics tools, and databases utilized in the two-by-two pipeline.

**Figure 2:**
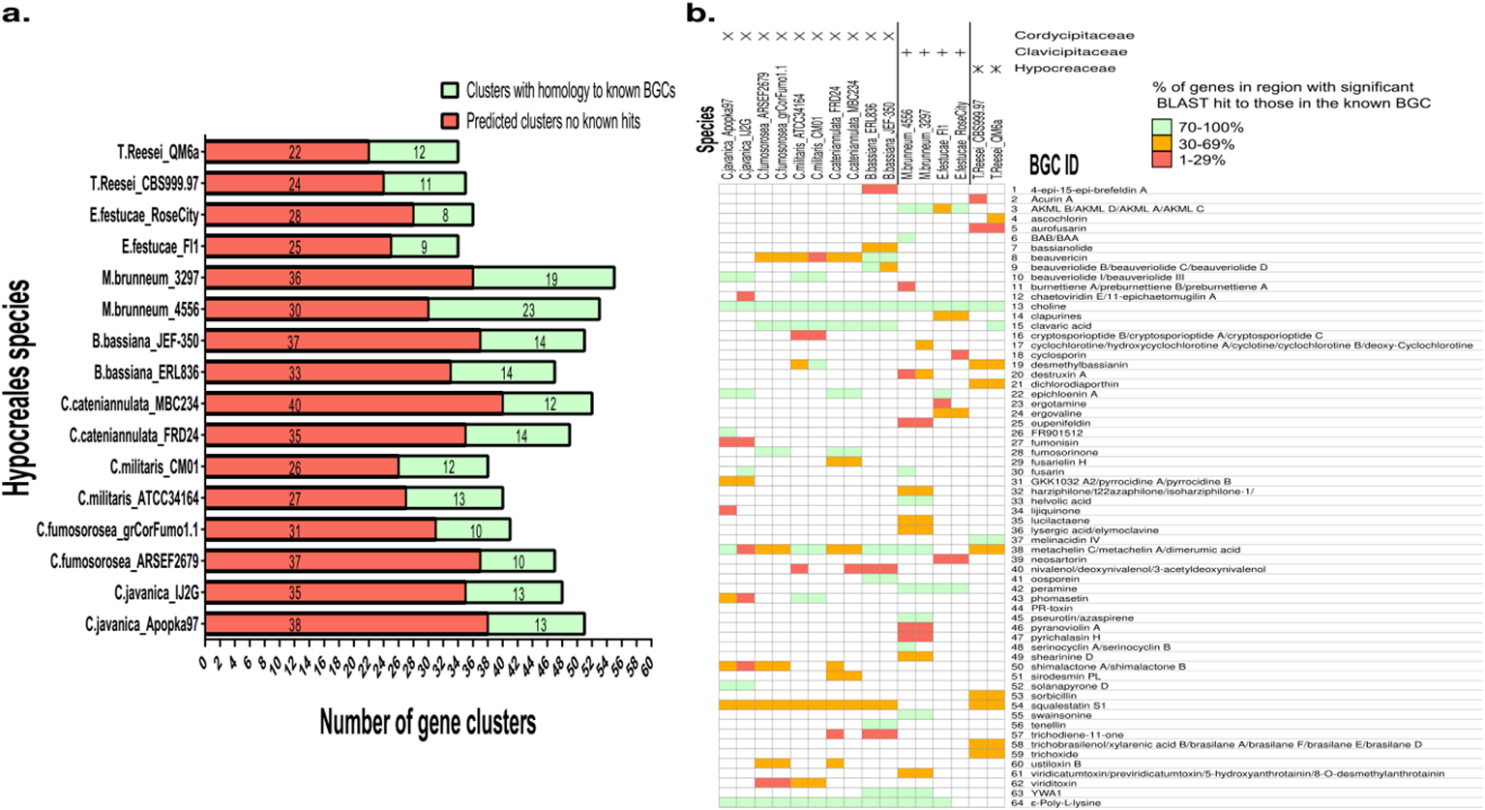
Two-by-two biosynthetic gene cluster analyses. **(a)** Biosynthetic gene clusters identified by AntiSMASH, showing the proportion of hits with homology to known gene clusters in the MIBiG database. **(b)** Distribution of gene clusters across fungal species and strains, showing binned percentage identity scores to known clusters in the MIBiG database.

The two-by-two approach used two strains from each species to perform *in-silico* biosynthetic gene cluster and orthogonal protein analyses against other species in the group (**Figure 1**). We performed an *in-silico* analysis using the fungal version of antiSMASH to quantify predicted biosynthetic gene clusters in each fungal species and to identify shared and species-specific clusters that had homology to known BGCs in the MIBiG database, providing a resource to support future research (**Figure 2**). *M. brunneum* exhibited the highest number of predicted biosynthetic gene clusters (BGCs), followed by *B. bassiana*, *Cordyceps cateniannulata*, and *C. javanica* (**Figure 2a**). Approximately one-third to one-quarter of the predicted gene clusters for each species had genes matched to known BGCs. Out of the 64 BGCs that had hits to known BGC clusters, 43 (67.2%) were unique to a single species within the Hypocreales fungi analyzed. For BGCs with known MIBiG database hits, we present a heat map showing the presence or absence across species and a color-coded key of the proportion of genes with significant BLAST matches in the predicted gene cluster (**Figure 2b**). The most prevalent gene cluster detected across all species of Hypocreales resembled that of the BGC for choline biosynthesis, a metabolite essential for fungal growth (Hai et al., 2019). Clusters resembling those of the siderophore metachelin C and antimicrobial poly-amino acid ϵ-poly-L-lysine were also detected in most fungal species analyzed (**Figure 2b**), and these compounds are known to be commonly produced by fungi (Giuliano Garisto Donzelli et al., 2015; Wang et al., 2021; Zhang et al., 2021; Pokhrel and Coleman, 2023).

Interestingly, a lower scoring hit (only some genes from the known cluster present) was observed for a gene cluster associated with the synthesis of the squalene-synthesis inhibiting compound squalestatin S1 within the Cordycipitaceae and Hypocreaceae families, but genes from this cluster were absent in the genomes of Clavicipitaceae species (Koulman et al., 2007; Bonsch et al., 2016). BGCs containing genes for the pathway synthesizing the steroid clavaric acid were also absent from species in the family Clavicipitaceae, and were also absent from *C. javanica* (Jayasuriya et al., 1998). Hits for genes resembling the genes in the cluster for fumosorinone synthesis, a compound found to inhibit protein tyrosine phosphatase 1B, a major negative regulator of the insulin signaling pathway, were detected in both species of *Cordyceps fumosorosea* from which the compound was first identified. Interestingly, we also detected a high homology hit in both *C. cateniannulata* genomes analyzed, to our knowledge, the first time this gene cluster has been reported in the species. A BGC resembling that of beauvericin, a cyclic hexadepsipeptide mycotoxin that has insecticidal, antimicrobial, antiviral, and cytotoxic activities (Wang and Xu, 2012), was detected in all genomes of species within Cordycipitaceae (with a high percent score in Beauveria species and lower percentage hits in Cordyceps species), but no genes for the cluster were detected in *C.javanica*. Genes resembling those found in the iron-binding siderophore epichloenin A (Koulman et al., 2012) were detectable in both genomes of *C.javania* and *C.cateniannulata*, but only present in one genome of *E. festucae*. Some of the genes for the known BGC synthesizing the neuritogenic polyketide Shimalactone A/B gene cluster (Sofiyev et al., 2008) were detected in the Cordyceps species excepting for *C.militaris* wherein no identity matches were detected. Genes matching those that synthesize the beauveriolide compounds were present in Cordyceps and Beauveria, as previously reported (Yin et al., 2020). Gene clusters that were uniquely identified in two strains of a single species and were known to be unique from that species (or at least absent from the other species analyzed) include; clusters resembling those of swainsonine, destruxin A and helvolic acid in *M. brunneum*, clusters resembling those of oosporein and tenellin in *B. bassiana*, clusters resembling those of melinacidin IV in *T. reesei*, and a cluster resembling that of ergovaline in *E. festucae*. Some predicted clusters had high percentage identity hits to known clusters, but were detected in only one of the two strains analyzed. These include clusters for; (2*Z*,4*E*,6*E*,10*E*)-9-hydroxydodeca-2,4,6,10-tetraenoic acid/(2*E*,4*E*,6*E*,10*E*)-9-hydroxydodeca-2,4,6,10-tetraenoic acid (BAA/BAB), serinocyclin A/serinocyclin B and fusarin only detected in *M. brunneum* 4556, epichloenin A only present in *E. festucae* Fl1, and clavaric acid only present in *T. reesei* QM6a. It is not clear whether these differences are due to a genuine gene variation between the two strains, or whether they are present in the genome of the second strain but not predicted due to sequencing and/or assembly issues. In this regard, it is worth noting that in the case of *M. brunneum*, the genome of 4556 is telomere length and was assembled using hybrid sequencing technology, whilst the 3297 genome was assembled as part of pioneering efforts to utilize massively parallel sequencing technologies on fungi for the first time (see dates, BUSCO scores and contig information in **Table 1**). Together, these results underscore the complexity of BGC prediction and demonstrate the value of the two-by-two approach in validating putative clusters through confirmation in at least two strains per species. *In-silico* BGC predictions cannot determine the compounds produced; functional validation requires knockout or heterologous expression, both non-trivial methods that benefit from better-validated clusters identified by the two-by-two approach.

### Two-by-two analyses of orthogonal proteins

**Figure 3:**
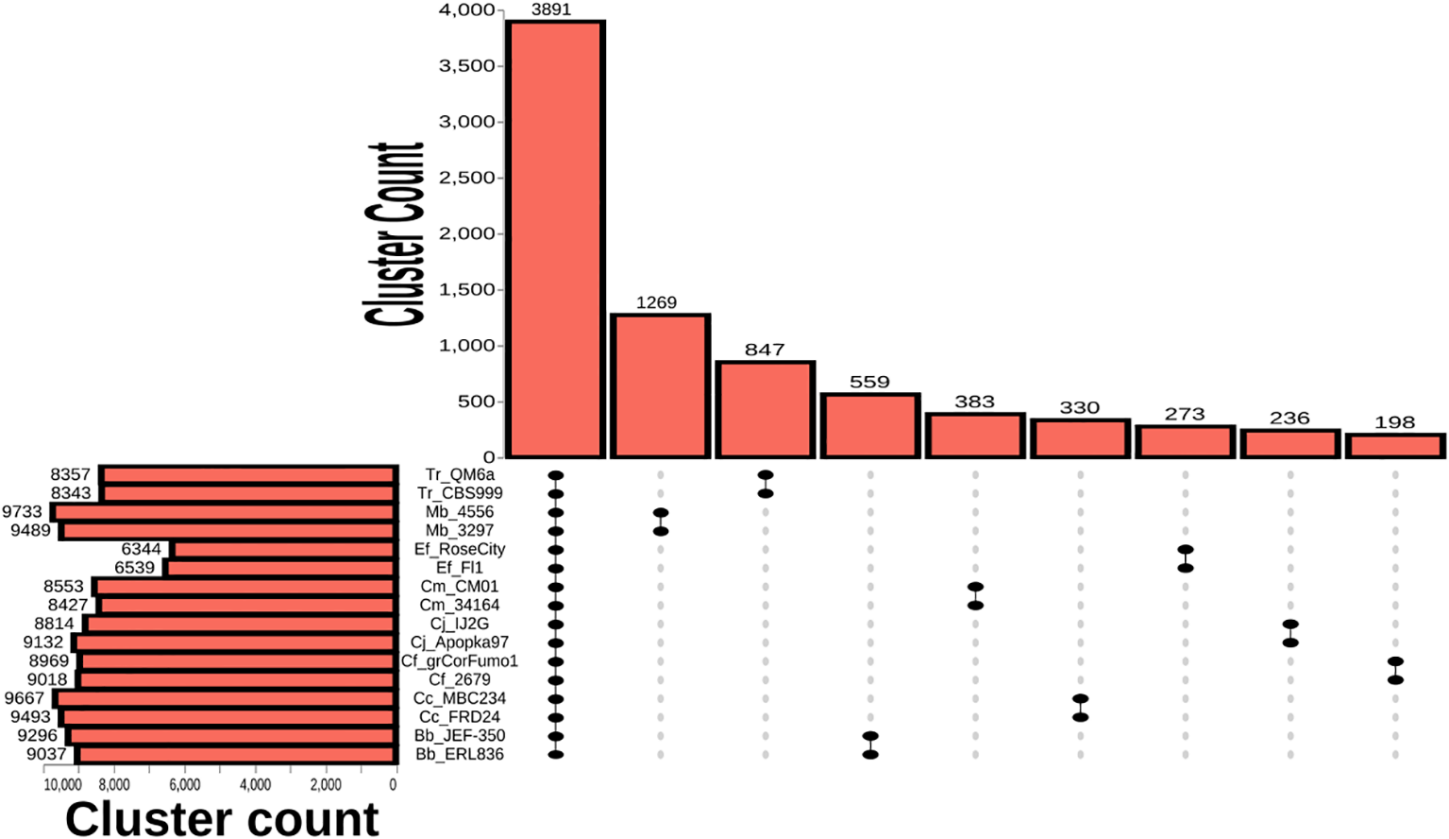
Two-by-two orthologous protein analysis of Hypocreales species. Vertical bars represent the counts of unique self-clusters between single species strains (two-by-two approach) and clusters containing proteins from all analyzed strains representing pan-Hypocreales genes. Horizontal bars represent the total number of clusters formed for each species.

To identify unique proteins in each species, orthologous protein analysis was performed with OrthoVenn3 using the OrthoFinder algorithm and using the two-by-two approach to identify strain self-clusters that were absent from other species (**Figure 3**).

A total of 3891 clusters were seen to contain orthologous proteins from the 16 strains comprising 8 species that were analyzed, and this represents the core genes for the group shared across all the species. The highest number of species self-clusters was observed between the two strains of *M. brunneum* analyzed (1269 clusters), followed by the two strains of *T. reesei* (847 clusters). *B. bassiana* formed 559 clusters, *C. militaris* 383 clusters, *C. cateniannulata* 330 clusters, *E. festucae* 273 clusters, *C. javanica* 236 clusters and *C. fumosorosea* 198 clusters (**Supplementary Table 1**). Singlet proteins that did not form a cluster with any other proteins ranged in number for each species from 1411 for *C. javanica* strain IJ2G to 56 for *M. brunneum* ARSEF 3297 with an average of around 250 proteins across all 16 strains analyzed (**Supplementary Table 2**). Clusters that were found to form uniquely within a single strain (strain self-orthologs) included; 21 clusters for *B. bassiana* JEF350, 17 clusters for *C. javanica* Apopka97, 11 clusters for *C. cateniannulata* MBC234, 9 clusters for *M. brunneum* ARSEF4556, 7 clusters for *C. cateniannulata* FRD24, 6 clusters for *C. javanica* IJ2G, 5 clusters for both *C. fumosorosea* grCorFumo1 and *B. bassiana* ERL836, 4 clusters for *E. festucae* Fl1, 3 clusters for *E. festucae* RoseCity, 2 clusters for *C. fumosorosea* ARSEF2679, 1 cluster for both *C. militaris* CM01 and *T. reesei* QM6a and no unique strain self-clusters were observed for *C. militaris* ATCC34164, *M. brunneum* ARSEF3296 or *T. Reesei* CBS999 (**Supplementary Table 3**).

Of the self-clusters that formed uniquely between two strains, 18 clusters were found to contain genes that produce 10 previously characterized natural products that have been demonstrated to be unique to the species (**Table 2**). These included: proteins responsible for synthesizing oosporein in *B. bassiana*, a metabolite required for fungal virulence that acts by evading host immunity to facilitate fungal multiplication in insects (Feng et al., 2015); proteins that produce the compound cordycepin in *C. militaris*, an adenosine derivative shown to have antibiotic, anti-inflammatory, and anticancer activities (Xia et al., 2017); the *Metarhizium* proteins Cuticle-degrading protease PR1 (St Leger et al., 1992) and various subtilisin-like proteins known to be involved in cuticle and collagen degradation (Bagga et al., 2004; Pannkuk et al., 2015), a known protein involved in the formation of appressoria (Zeng et al., 2018), and various proteins involved in the production of swainsonine (Cook et al., 2017), a cytotoxic fungal alkaloid and a potential cancer therapy drug. In the non-entomopathogenic fungal species, a protein known to take part in synthesis of sorbicillinoids was detected in *T. reesei*- an anti-fungal metabolite known to be produced by this species (Derntl et al., 2017). Two proteins known to be involved in the synthesis of the antifungal verlamelin were detected in *E. festucae* (Wang et al., 2019). Genes for oosporein, sorbicillin, and swainsonine were also detected in the BGC analyses (**Figure 2c**), however, it was encouraging to see that multiple proteins in this pathway were also detected as unique species self-clusters in the orthogonal protein analysis pipeline, validating the approach.

**Table 2:** Species-specific self-clusters identified via the two-by-two approach with previously characterized unique functions.

Species	Cluster ID	Number of proteins in cluster	Swiss-prot hit (Entry - species)	Swiss-prot hit protein name	Members of cluster	Function	Reference
*B.bassiana*	cluster10663	3	OPS1 - Beauveria bassiana	Orsellinic acid synthase	Bb_ERL836|KAF1732856.1; Bb_JEF-350|KAH8713103.1; Bb_JEF-350|KAH8713104.1	Gene cluster that mediates the biosynthesis of the bibenzoquinone oosporein, a metabolite required for fungal virulence that acts by evading host immunity to facilitate fungal multiplication in insects	(Feng et al., 2015)
	cluster11284	2	OPS7 - *Beauveria bassiana*	Oxidoreductase OpS7	Bb_ERL836|KAF1732862.1; Bb_JEF-350|KAH8713097.1		
	cluster11283	2	J5J924 - Beauveria bassiana	MFS transporter OpS2	Bb_ERL836|KAF1732858.1;Bb_JEF-350|KAH8713102.1		
	cluster11282	2	OPS3 - *Beauveria bassiana*	Oosporein cluster regulator OpS3	Bb_ERL836|KAF1732857.1; Bb_JEF-350|KAH8713101.1		
*C. militaris*	cluster12734	2	No hit		Cm_34164|ATY65128.1; Cm_CM01|EGX93064.1	Product encoded by gene CNS1 of the Cordycepin biosynthetic gene cluster	(Xia et al., 2017)
	cluster12735	2	O05502 - *Bacillus subtilis*	Uncharacterized protein YdhJ	Cm_34164|ATY65129.1; Cm_CM01|EGX93065.1	Product encoded by gene CNS2 of the Cordycepin biosynthetic gene cluster	
	cluster12736	2	No hit		Cm_34164|ATY65134.1; Cm_CM01|EGX93066.1	Product encoded by gene CNS3 of the Cordycepin biosynthetic gene cluster	
*M. brunneum*	cluster13200	2	P29138 - *Metarhizium anisopliae*	Cuticle-degrading protease PR1	Mb_3297|KID60942.1; Mb_4556|QLI68484.1	Cuticle-degrading protease, Chymoelastase, PR1. Capable of breaching the insect cuticle	(St Leger et al., 1992)
	cluster13207	2	L8G6I7 - *Pseudogymnoascus destructans*	Subtilisin-like protease 1	Mb_3297|KID61215.1; Mb_4556|QLI74370.1	Major secreted subtilisin-like serine endopeptidase. Degrades the nonhelical regions of collagen that function in the cross-linking of the helical components	(Bagga et al., 2004; Pannkuk et al., 2015)
	cluster13386	2	L8GD75 - *Pseudogymnoascus destructans*	Subtilisin-like protease 3	Mb_3297|KID66974.1; Mb_4556|QLI70477.1	Major secreted subtilisin-like serine endopeptidase. Degrades the nonhelical regions of collagen that function in the cross-linking of the helical components	(Bagga et al., 2004; Pannkuk et al., 2015)
	cluster13323	2	A0A0B4GDU5 - *Metarhizium brunneum*	Scytalone dehydratase-like protein Arp1	Mb_3297|KID65390.1; Mb_4556|QLI69143.1	Scytalone dehydratase-like protein; part of the Pks2 gene cluster that mediates the formation of infectious structures (appressoria), enabling these fungi to kill insects faster	(Zeng et al., 2018)
	cluster13990	2	D4AX50 - *Arthroderma benhamiae*	Subtilisin-like protease 8	Mb_3297|KID76020.1; Mb_4556|QLI67847.1	Secreted subtilisin-like serine protease with keratinolytic activity that contributes to pathogenicity	(Bagga et al., 2004)
	cluster10467	4	E9F8M0 - *Metarhizium robertsii*	Transmembrane transporter swnT	Mb_3297|KID64719.1; Mb_3297|KID73970.1; Mb_4556|QLI68254.1; Mb_4556|QLI74702.1	Gene cluster that mediates the biosynthesis of swainsonine, a cytotoxic fungal alkaloid and a potential cancer therapy drug.	(Cook et al., 2017)
	cluster13587	2	D4AU27 - *Arthroderma benhamiae*	Swainsonine transporter swnT	Mb_3297|KID72725.1; Mb_4556|QLI71645.1		
	cluster10517	4	D4AU26 - *Arthroderma benhamiae*	Dioxygenase swnH1	Mb_3297|KID73870.1; Mb_3297|KID73969.1; Mb_4556|QLI67169.1; Mb_4556|QLI67423.1		
	cluster13740	2	E9F8M4 - *Metarhizium robertsii*	Dioxygenase swnH2	Mb_3297|KID73974.1; Mb_4556|QLI68135.1		
*T. reesei*	cluster14860	2	G0R6S7 - *Trichoderma reesei*	Short chain dehydrogenase sor7	Tr_CBS999|TreeseiCBS999.97_006656-T1; Tr_QM6a|TreeseiQM6a_006735-T1	Short chain dehydrogenase; part of the SOR gene cluster that mediates the biosynthesis of sorbicillinoids, a diverse group of yellow secondary metabolites that restrict growth of competing pathogenic fungi but not of bacteria.	(Derntl et al., 2017)
*E. festucae*	cluster12871	2	A0A024FA41 - *Lecanicillium* sp	Fatty acid hydroxylase vlmA	Ef_Fl1|QPG93928.1; Ef_RoseCity|EfestucaeRoseCity_000440-T1	Fatty acid hydroxylase; part of the gene cluster that mediates the biosynthesis of verlamelin, a lipopeptide that exhibits antifungal activity against plant pathogenic fungi. This gene product has previously been observed in *E. festucae* and has been detected in *E. festucae* infected *Festuca rubra*	(Wang et al., 2019)
	cluster13039	2	A0A024F910 - *Lecanicillium* sp	Nonribosomal peptide synthetase vlms	Ef_Fl1|QPH06327.1; Ef_RoseCity|EfestucaeRoseCity_000031-T1		

BGC algorithms such as antiSMASH have previously been shown to be unable to detect the cordycepin *Cns* cluster (Hong et al., 2024), and we were also unable to detect it using the latest version of this tool but were able to detect 3 of the 4 genes in the cluster using the two-by-two orthologous protein analysis (**Table 2**). Given that the two-by-two approach was able to detect this gene cluster that could not be detected by BGC identification algorithms, we mapped all the species self-cluster proteins for fungi with telomere length genome assemblies back to their respective genomes, and then compiled a list of potential gene clusters by mapping genes unique to each species that were within 5kb of each other in the genome (**Supplementary Table 4**). We present this list as a resource that likely contains other novel BGCs for these species that could not be detected by antiSMASH. Overall, the two-by-two approach when used for individual proteome analysis effectively identified known distinct proteins for each species within the group, unaffected by the degree of relatedness or genetic distance among the species analyzed and, compared to the BGC analysis, was seemingly less affected by the quality of the genomes utilized.

### Identification of a fungal Tc toxin complex and functional myco-serpin

Next, we assessed self-cluster hits that were found to contain proteins with high homology to previously characterized proteins that are involved in pathogenesis, antibiotic production, or distinctive catabolic processes, and we have listed these in **Supplementary Table 5**.

**Figure 4:**
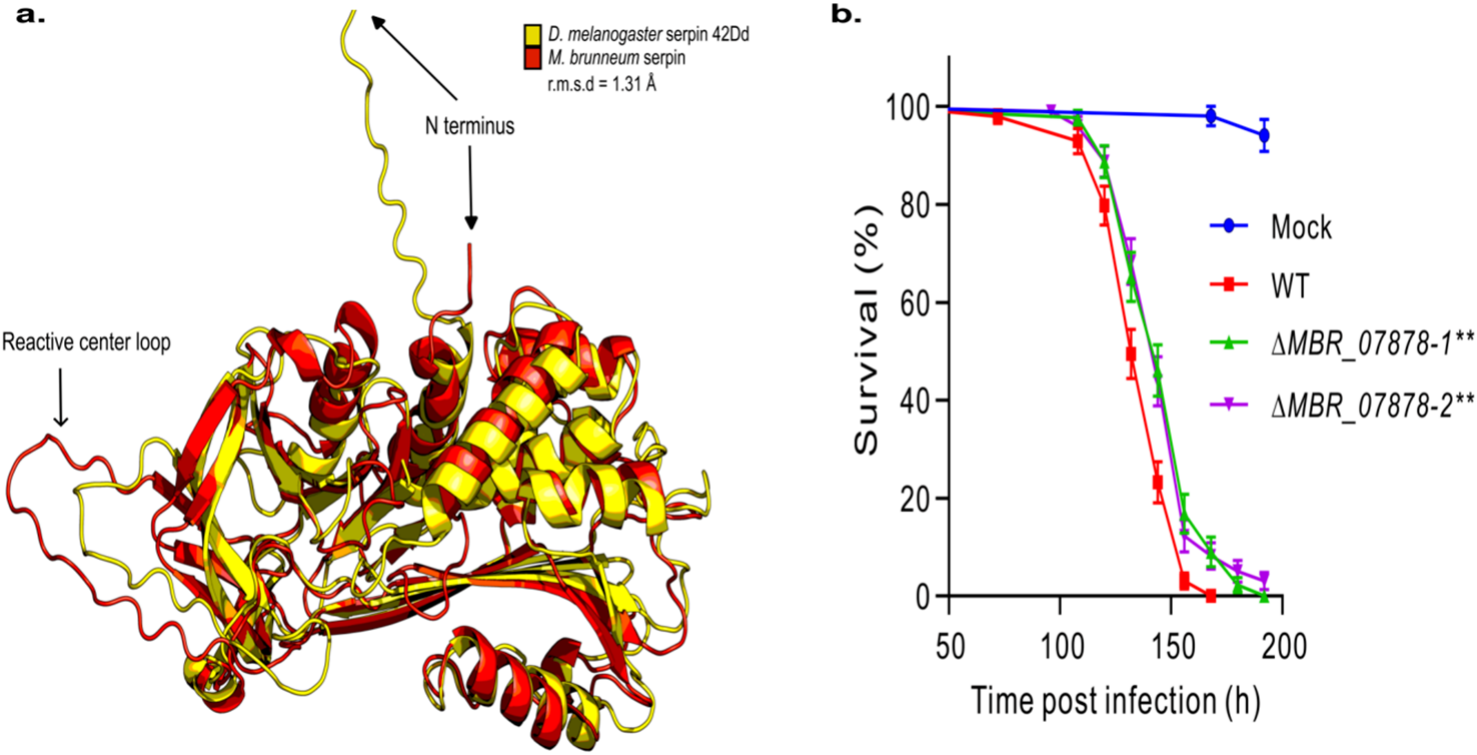
Identification of a novel fungal myco-serpin. **(a)** Structural homology between the *Metarhizium brunneum* serpin and Spn44Dd (UniProt: Q7YTY6) of *Drosophila melanogaster*, a negative regulator of the melanization and Toll pathways. **(b)** Survival plot of *Drosophila* females infected with the *M. brummeum* wild-type (WT) and serpin gene-knockout mutants. Panel **(b)** mock control flies were treated with Tween 20. Survival differences between WT and individual mutants were determined by log-rank test: **P < 0.01.

Interestingly, we observed a previously undescribed protein in *M. brunneum* with homology to serpin 42Dd of the fruit fly, *Drosophila melanogaster* (cluster 13612, ORF G6M90_00g004700 of *M. brunneum* 4556). The protein was predicted to have a classical serpin structural domain (IPR023796) spanning from amino acids 13–365 of the 365 amino acid sequence. A structural alignment revealed close structural homology between the two proteins and the predicted protein structure was found to have the classical conserved serpin folds and a reactive center loop (**Figure 4a**). This protein was not present in any of the other entomopathogenic fungi, but a blast search revealed it to be present in multiple species of *Trichophyton*, a mammalian pathogen, as well as being present in multiple species of *Aspergillus* and *Fusarium*, which contain species that are opportunistic mammalian pathogens. To determine the potential function of this gene in fungal virulence, a gene knockout line of this serpin, designated MBR_07878, was made. Topical infection of *D. melanogaster* revealed that the virulence of null mutants was significantly impaired (log-rank test, P < 0.01) compared to the wild-type strain (**Figure 4b**), thus implicating the protein in the infection process. By 132 hours post-infection (hpi), the wild-type strain exhibited significantly higher virulence than the mutant strains, with survival rates of approximately 50% and >60%, respectively. Furthermore, while the wild-type strain achieved 100% mortality by 168 hpi, approximately 10% of the host population remained viable following infection with the mutant strains.

**Figure 5:**
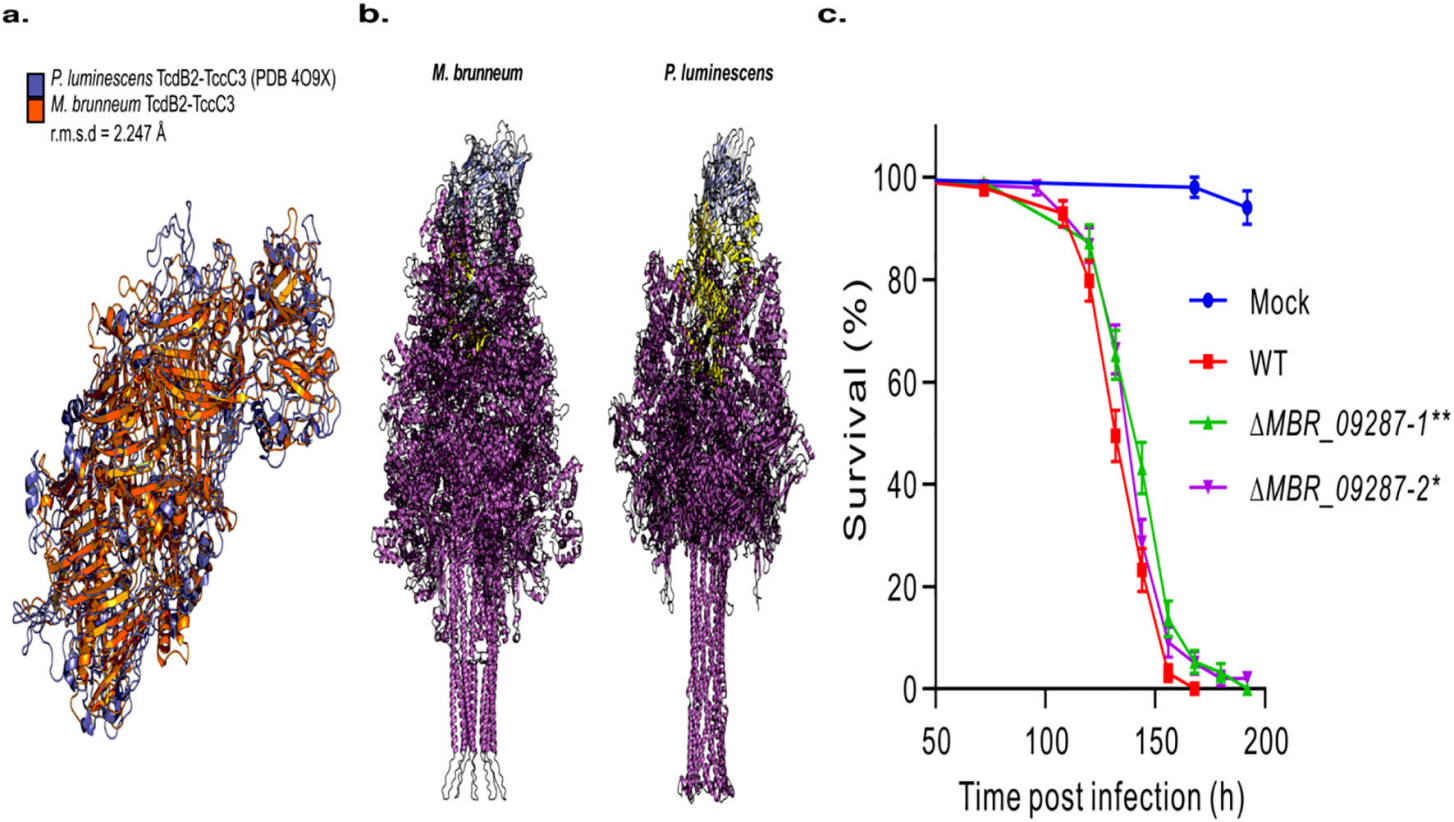
Identification of a fungal Tc toxin complex. **(a)** Structural overlap of the *Metarhizium brunneum* 4556 Alphafold3 docked YenC1 and YenB proteins with the TcdB2 and TccC3 toxin subunits of *Photorhabdus luminescens* (PDB:4O9X). **(b)** Structure of *in-silico* assembled *Metarhizium brunneum* 4556 Tc toxin complex and a Cryo-EM structure of *Photorhabdus luminescens* ABC holotoxin assembly embedded in a lipid nanodisc (PDB:6SUF). The pentameric TcdA subunits are colored magenta, the TcdB subunit is colored yellow and the TcdC subunit is colored light blue. **(c)** Survival plot of *Drosophila* females infected with the *M. brunneum* wild-type and Tc toxin gene mutants. Panel **(c)** mock control flies were treated with Tween 20. Survival differences between WT and individual mutants were determined by log-rank test: *P < 0.05; **P < 0.01.

Another hit in the *M. brunneum* species self-cluster showed a hit to subunit C1 of the Tc toxin complex of *Yersinia entomophaga* (cluster 10481, ORF G6M90_00g045720 of *M. brunneum* 4556). Tc toxins complexes are virulence factors of many infectious and entomopathogenic bacteria (Roderer and Raunser, 2019). The toxins are composed of three subunits that assemble to perforate a host membrane and translocate a toxic enzyme into the cell (Roderer and Raunser, 2019). To date, no such toxin complex has been described in a fungal species. The *Y. entomophaga* Tc toxin subunit C1 ortholog found in Metarhizium mapped to chromosome 2 (*M. brunneum* strain 45556- regions 5382531-5386727). This 1258 amino acid protein was found to contain an Insecticide toxin TcdB middle/C-terminal region domain (IPR022044). Interestingly, a downstream neighboring (*M. brunneum* strain 45556- regions 5387051-5388929) gene was found to have homology to *Y. entomophaga* Tc toxin subunit B (ORF G6M90_00g045730 of *M. brunneum* 4556). Using AlphaFold3, we computationally docked the *M. brunneum* toxin subunits B and C (**Figure 5a**). The predicted multimer showed good structural homology to a crystal structure of TcdB2-TcdC3 subunits of the bacteria *Photorhabdus luminsecens* [PDB:4O9X (Meusch et al., 2014)], with an RMSD of 2.247 Å (a measure of structural divergence). This metric represents the spatial average of the distances between equivalent atom pairs in the superimposed structures, where a lower RMSD value indicates higher structural conservation, and a value of less than 2.0 Å showing excellent similarity.

We then conducted a protein blast of the *M. brunneum* proteome using the sequence for *Yersinia Entomophaga* Tc toxin subunits A1 and A2. A hit was located for subunit A2 that mapped to chromosome 4 of *M. brunneum* 4556 (regions 1096054-1104757). This protein was found to be 2882 amino acids long and contained a Tc toxin complex TcA C-terminal TcB-binding domain (IPR040840) at positions 2440-2726, a Neuraminidase-like domain (IPR041079) at positions 1495-1655, and an ABC toxin N-terminal region domain (IPR046839) at positions 1344-1465 (ORF G6M90_00g069900 of *M. brunneum* 4556). A blast search of this protein confirmed its presence in multiple other *Metarhizium* species including; *M. robertsii*, *M. anisopliae*, *M. humberi*, *M. guizhouense*, and *M. album*. Previous studies in bacteria have demonstrated the A subunit alone is readily toxic but its effect is enhanced with the other two subunits docked (Waterfield et al., 2005). We used AlphaFold3 to predict structures of the various subunits of the *M. brunneum* Tc toxin complex and input these smaller subunits into CombFold to generate the 16,253 amino acid toxin complex and compared it to the a Cryo-EM structure of *Photorhabdus luminescens* ABC holotoxin assembly embedded in a lipid nanodisc (**Figure 5b**). Both toxin complexes have the distinct common architecture of Tc toxins. An A2 subunit Tc toxin gene knockout *M. brunneum* strain (MBR_09287) was made, which showed an increased survival rate for the null mutants when compared to wild-type when after topical challenge of *D. melanogaster* (**Figure 5c**), thus implicating the protein in the infection process. By 132 hours post-infection (hpi), the wild-type strain exhibited significantly higher virulence than the mutant strains, with survival rates of approximately 50% and >60%, respectively. Furthermore, while the wild-type strain achieved 100% mortality by 168 hpi, >5% of the host population remained viable following infection with the mutant strains. The identification of these previously unreported functional genes highlights the utility of the two-by-two approach in discovering novel functional genes.

## Discussion

We put forward a simple, novel, validated approach to determine the unique genes more accurately within a pre-selected group of fungi and is capable of distinguishing genes that are unique to a strain. We validated the method by selecting a group of fungi that have been subjected to decades of wet-lab experimentation, and confirmed that the approach could successfully detect genes and gene clusters that have previously been demonstrated to be unique to certain species within the group. Using the two-by-two approach combined with in silico BGC prediction, we generated a heatmap depicting the biosynthetic potential of species in the families Cordycipitaceae, Clavicipitaceae, and Hypocreaceae within the order Hypocreales, providing a framework for future functional and comparative BGC analyses in these and related fungi. Two-by-two analyses of BGC clusters revealed that strains within a species harbored similar numbers of total BGCs, with a general trend for predicted BGCs to be shared between strains, irrespective of genome quality. For instance, the two *C.javanic* strains analyzed differed markedly in genome quality (173 scaffolds and 658 contigs for the IJ2G strain versus seven complete chromosomes for the Apopka 97 strain), yet the total numbers of predicted BGCs were similar (48 and 51, respectively). Of the 15 BGCs with matches in the MIBiG database, 11 were shared by both strains, while the remaining four were strain-specific—two unique to Apopka 97 and two unique to IJ2G. Moreover, the *B. bassiana* strains differed in protein BUSCO scores (91.3% versus 95.5%), a standard metric of genome completeness, yet all predicted BGCs with matches to known clusters in the MIBiG database were recovered in both strains. The ability of the fungal version of antiSMASH to predict BGCs appeared largely unaffected by genome quality. In *T. reesei*, where genome quality metrics were comparable between strains (seven complete chromosomes and BUSCO scores of 98.0 and 98.7), certain BGC predictions were strain-specific—for instance, a high-quality match to clavaric acid was detected only in strain QM6a. These findings underscore the complexity of BGC evolution in *Hypocreales* fungi and highlight the value of two-by-two analyses for robust identification of putative BGCs prior to resource-intensive functional studies, as they increase confidence that a BGC is truly present in multiple strains of a species. A further limitation is that differences in algorithm parameters—for example, running the tool with relaxed versus strict settings—can produce varying results, further complicating comparisons across studies. Integrating our current framework with multi-omic approaches (transcriptomics and metabolomics) would provide a more robust characterization of the infection process and facilitate the precise identification of alternative splice variants. However, a significant limitation is that key virulence genes may remain transcriptionally silent under *in vitro* conditions. Conversely, *in vivo* analyses are inherently complicated by the high background of host-derived transcripts and metabolites, which can interfere with the detection of fungal-specific signals.

We performed two-by-two orthogonal protein analyses using protein sets that were either deposited in GenBank, when available, or predicted in silico in this study. We showed that among orthologous proteins that uniquely self-clustered between two strains, we could identify proteins known to be species-specific (listed in **Table 2**), thereby validating the technique’s ability to detect genuinely unique genes in species. Building on this validation, we compiled a list of orthologous proteins that uniquely self-cluster between two strains of the same species that have high sequence homology to previously characterized proteins known to have roles in pathogenesis, antibiotic production, or distinctive catabolic processes (**Supplementary Table 5**). **Supplementary Table 1** contains a full list of self-clusters between two strains of each species analysed in this study. Additionally, we compiled a list of singlet proteins that did not form any orthologous clusters (**Supplementary Table 2**), as well as a list of proteins that formed clusters exclusively with proteins from the same strain (**Supplementary Table 3**). We also mapped genes unique to each species that were located within 5 kb of one another in the genome (**Supplementary Table 4**); these may represent novel gene clusters not detected by antiSMASH, as exemplified by the cordycepin BGC (Hong et al., 2024). These resources can be used for bioprospecting novel genes and gene clusters within these species, and may also serve to subtract common fungal genes or clusters when analyzing species outside our study group. Given the gene richness of fungal genomes, declining sequencing costs, and improving genome assembly algorithms, we anticipate that comparative genomics, including our two-by-two approach, will continue to enhance fungal gene discovery. This method complements RNA-Seq by simplifying differential gene expression analyses and identifying species-specific genes irrespective of their expression, thereby facilitating the discovery of novel cryptic genes.

Two *M. brunneum* self-clusters represent novel genes that have never been described before in fungi. We have identified the first functional myco-serpin that has significant homology to a known *D. melanogaster* immunomodulatory serpin. A major function of serpins in *Drosophila* is to confine immune reactions and prevent excessive activation (Levashina et al., 1999; Shan et al., 2023). Serpin 42Dd is a negative regulator of the Toll and melanization cascade pathways, blocking the serine proteases ModSP and Grass, with mutations in its gene leading to spontaneous melanization and constitutive activation of the Toll pathway (Shan et al., 2023). A recent study has shown that *Metarhizium robertsii* produces an immune modulating protein during infection that can also block the Toll pathway (Lu et al., 2024). The Metarhizium serpin may play a role in immune evasion, as previous studies have shown that a serpin-like compound in the Microsporidia, *Nosema bombycis*, can suppress insect host hemolymph melanization (Bao et al., 2019). Parasitic insects have also been shown to use serpin-like proteins to dampen the melanization response against eggs they inject into other insects (Asgari et al., 2003), and poxviruses produce secreted serpin-like proteins to dampen inflammatory responses against mammals (Nash et al., 1997). Furthermore, a serpin-like protein from a symbiotic human gut bacteria has been shown to protect against exogenous proteolysis by gut proteins (Ivanov et al., 2006). Moreover, the serpin shares homology with other previously undescribed fungal serpins in *Fusarium*, *Aspergillus*, *Microsporum* and *Trichophytan*, with the two former species being opportunistic human pathogens and the latter two being parasitic mammalian pathogens. It would be of great interest to assess the impact on infection of knocking out the gene from a mammalian parasite, as mammals are also known to utilize serpins to regulate the immune system. From the gene knockout mutant, we cannot exclude the possibility that the serpin acts on a fungal serine protease, as proteins can exhibit pleiotropic effects; however, no detrimental impact on fungal growth was observed in the knockout strain. Prior to this study, the only other fungi to have been reported to have a serpin-like protein is the anaerobic fungus *Piromyces* sp., which contains a multi-domain protein that forms part of the cellulosome and contains a smaller serpin-like domain (Steenbakkers et al., 2008).

Finally, utilizing the two-by-two approach to analyze orthogonal proteins, we show that *M. brunneum* contains multiple subunits of a Tc toxin complex that exhibit significant structural homology to the well-characterized bacterial Tc toxin complexes (Gatsogiannis et al., 2013; Leidreiter et al., 2019; Roderer et al., 2020; Song et al., 2021; Belyy et al., 2022; Xu et al., 2022; Sitsel et al., 2024). Two of these subunits were found to be neighboring one another on the same chromosome, whilst the third subunit was found on a separate chromosome. The composition of this protein complex and its similarity to bacterial Tc toxins may indicate acquisition by fungi through horizontal gene transfer, a well-documented process (Bruto et al., 2014; Habig et al., 2024). The fact that the two subunits are located on chromosome 2, whilst the third is located on chromosome 4 may be attributable to a peculiar form of chromosomal evolution termed mesosynteny that has only been reported in Ascomycete fungi (Hane et al., 2011). The serpin and Tc complex are upregulated in *Metarhizium brunneum* during infection of *Myzus persicae*, as revealed by a recent transcriptomic study of differentially expressed genes [in **Supplementary Figure 1** of reference (Reingold et al., 2024)]. Further work should assess the functional activity of the fungal toxin complex in purified form to assess its efficacy in comparison to bacterial Tc toxin complexes. Since the fungal proteins were predicted from spliced genes, investigating the potential enhancements in the fungal protein upon expression would be intriguing. Given the recent recognition of horizontal gene transfer from bacteria to fungi (Bruto et al., 2014), it would be interesting to investigate whether splicing of these acquired genes can generate enhanced protein variants, potentially representing a novel mechanism of protein evolution.

In this study, we have limited our search to truly unique genes between species by looking at strain self-clusters, however, the approach would also allow one to determine interesting genes shared by two very closely related species but potentially not the rest of the species within a test group (e.g. by looking at clusters formed between two paired strains from 2 separate species). The species selected for analyses will likely affect the outputs, however, genes/gene clusters that are truly unique to only one species of fungi will likely always form strain self-clusters, regardless of what other fungi are added to the group. This was underscored by our finding that the serpin protein identified in Metarhizium is absent from other fungi within this group, yet present in more distantly related lineages. We envision that the approach can be used to elucidate unique genes in organisms from other kingdoms that have genome architecture and species diversity comparable to fungi.

## Methods

### Gene predictions and annotations

Biosynthetic gene cluster predictions were performed using the fungal version of the antiSMASH web server (v7.1) (Blin et al., 2023), setting the detection strictness to ‘relaxed’ and activating all extra features. The presence of signal peptides was predicted using SignalP 5.0 (Almagro Armenteros et al., 2019). Protein domain analyses was performed using InterProScan (v5.65-97.0). FunAnnotate (v1.8.15) was used to predict and annotate genes for the Hypocreales fungal genomes used in this study that lacked gene predictions in Genbank. BUSCO analyses were performed with BUSCO version 5.5.0 (Simão et al., 2015), using the hypocreales_odb10 lineage gene set. To determine the core genes shared across the Hypocreales species analyzed, comparison of orthologous gene clusters between the protein sets for each of the Hypocreales fungi were performed with a standalone version of OrthoVenn3 using the OrthoFinder algorithm (Sun et al., 2023). The AlphaFold3 server was used to predict protein structure and dock multimers (Abramson et al., 2024). Overlaps of protein structure were performed using the align tool in PyMOL Version 3.0 (The PyMOL Molecular Graphics System, Version 3.0). *In-silco* structural prediction of the *M. brunneum* 4556 was Tc toxin complex was performed using CombFold (Shor and Schneidman-Duhovny, 2024), using multiple AlphaFold3 docked dimer combinations as input.

### Gene deletions and fly survival assays

Deletion of the serpin and Tc genes were conducted in *M. brunneum* by homologous replacement (Lu et al., 2024). In brief, the 5’- and 3’-flanking regions of each gene were amplified by PCR using different primer pairs (**Supplementary Table 6**), and the purified products were cloned into the binary vector pDHt-bar (conferring resistance to ammonium glufosinate) (Lu et al., 2024). Constructs were individually transformed into the *Agrobacterium tumefaciens* AGL1 strain, which was used to infect the wild-type spores of *M. brunneum*. The drug-resistance colonies were selected and verified by PCR analysis. At least, two independent mutant isolates were selected for survival assays against the females of *D. melanogaster* (3 days post eclosion). Two-week-old conidia of the wild-type and mutants were harvested from the potato dextrose agar, and suspended in 0.01% Tween-20. The spore suspensions were adjusted to 5 ×10^5^ conidia/ml, and used for topical infection of female flies (Lu et al., 2024). Flies treated with Tween 20 carrier were included as a mock control. There were more than 70 flies used for each treatment, and insect survivals were recorded every 12 hours. The difference between wild-type and mutant strains was examined by Kaplan-Meier analysis utilizing the log-rank test using GraphPad Prism version 10.1 (GraphPad software Inc., San Diego, CA).

### Data and materials availability

Output files have been deposited in the following GitHub repository - https://github.com/zacksaud/Cordyceps-javanica-Assembly-Project. The following genomes and/or information on the genome assemblies were retrieved from NCBI’s GenBank database; Beauveria bassiana ERL836 (accession number: GCA_010099065.1), *Beauveria bassiana* JEF-350 (accession number: GCA_021365345.1), *Cordyceps javanica* IJ2G (accession number: GCA_006981975.1), *Cordyceps javanica* Apopka 97 (accession number: GCA_051103115.1) *Cordyceps fumosorosea* ARSEF 2679 (accession number: GCA_001636725.1), *Cordyceps fumosorosea* grCorFumo1 (accession number: GCA_963580265.1), *Cordyceps militaris* ATCC 34164 (accession number: GCA_008080495.1), *Cordyceps militaris* CM01 (accession number: GCA_000225605.1), *Cordyceps cateniannulata* FRD 24 (accession number: GCA_028828415.1), *Cordyceps cateniannulata* MBC 234 (accession number: GCA_030411495.1), *Metarhizium brunneum* ARSEF 4556 (accession number: GCA_013426205.1), *Metarhizium brunneum* ARSEF 3297 (accession number: GCF_000814965.1), *Epichloe festucae* Fl1 (accession number: GCA_003814445.1), *Epichloe festucae* RoseCity (accession number: GCA_016859245.1), *Trichoderma reesei* CBS999.97 (accession number: GCA_016806755.1), *Trichoderma reesei* QM6a (accession number: GCA_002006585.1).

## Data Availability

The datasets presented in this study can be found in online repositories. The names of the repository/repositories and accession number(s) can be found in the article/**Supplementary Material**.
